# Targeted next-generation sequencing confirms rabies in a patient with unknown exposure: a case report

**DOI:** 10.3389/fmed.2026.1766261

**Published:** 2026-04-01

**Authors:** Runqi Li, Chenmiao Jin, Zhongqi Zhang, Chen Zhang, Xinlong Chen

**Affiliations:** 1 Department of Critical Care Medicine, Affiliated Hospital of Nantong University, Nantong, Jiangsu, China; 2 School of Medicine, Nantong University, Nantong, Jiangsu, China; 3 Department of Medicine, Dinfectome Inc., Nanjing, Jiangsu, China

**Keywords:** atypical presentation, bronchoalveolar lavage fluid, critical care, *Lyssavirus*, rabies, targeted next-generation sequencing (tNGS)

## Abstract

This report describes a confirmed rabies case with no clear exposure history. A 68-year-old female was hospitalized due to chest tightness and shortness of breath, subsequently developing typical symptoms including agitation, photophobia, and hydrophobia, requiring transfer to the ICU. The family denied any animal bite history, and physical examination revealed only superficial marks on the left knee. Probe-capture targeted next-generation sequencing (tNGS) of bronchoalveolar lavage fluid (BALF) detected 279 rabies virus sequences, confirmed by Sanger sequencing. The patient ultimately succumbed to circulatory and respiratory failure after the family withdrew life support. This case demonstrates that probe-capture tNGS can serve as an effective tool for diagnosing rabies in living patient without typical exposure using BALF samples.

## 1 Introduction

Rabies is a fatal zoonotic infectious disease caused by the rabies virus (RABV), a member of the genus *Lyssavirus*, family Rhabdoviridae, with high neurotropism and nearly 100% case fatality once symptoms manifest (1–3). In China, most cases occur in rural areas, with dogs accounting for the majority of transmission (4). A persistent challenge is that many cases are diagnosed clinically, while laboratory confirmation rates remain low, delaying early diagnosis and intervention (5). Diagnostic systems have limitations: the direct fluorescent antibody test (dFAT) requires brain tissue and is not applicable intravitam; RT-PCR is a commonly used method, but the sensitivity is affected by sample types (6).

Unbiased sequencing approaches (mNGS) have identified RABV in atypical presentations or unclear exposure histories (7, 8). Targeted next-generation sequencing (tNGS), based on multiplex PCR or probe-hybridization capture, has emerged with advantages of lower host background, reduced cost, and combined DNA/RNA pathogen detection in a unified workflow (9–11). We present a rabies case diagnosed intravitam by probe-capture tNGS of bronchoalveolar lavage fluid (BALF) in a patient with unverified exposure and atypical early manifestations, supporting the utility of tNGS for elusive pathogens such as RABV.

## 2 Case presentation

**FIGURE 1:**
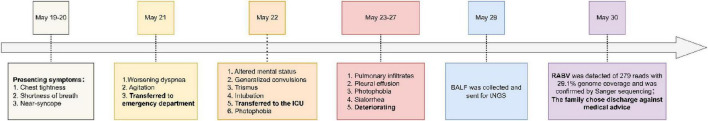
Timeline of key clinical events and diagnostics in a patient with rabies confirmed by BALF-based tNGS. The patient presented with a feeling of chest tightness, accompanied by acute shortness of breath and episodes of transient vision loss (May 20). The patient’s dyspnea progressively worsened and was accompanied by marked agitation, further was urgently transferred to the Emergency Department (May 21). The patient was intubated and transferred to the ICU following the acute onset of impaired consciousness, generalized seizures with trismus, and was found to be photophobic (May 22). The patient exhibited progressive pulmonary infiltrates, pleural effusion, photophobia, and sialorrhea. This multi-system deterioration prompted escalation of care (May 23–26). BALF was collected and sent for tNGS analysis (May 27). The presence of RABV, with 279 aligned reads providing 29.1% coverage of the viral genome was revealed and validated using Sanger sequencing. The patient’s family elected for discharge against medical advice (May 28). BALF, bronchoalveolar lavage fluid; tNGS, targeted next-generation sequencing; RABV, rabies virus.

A 68-year-old female patient presented with persistent precordial chest tightness of unknown cause, accompanied by shortness of breath, amaurosis fugax, and nausea (without vomiting), starting on May 19. She sought medical attention at a local hospital. A concise timeline of the clinical course and key diagnostic events is shown (Figure 1 and Supplementary Table 1). An electrocardiogram (ECG) revealed sinus tachycardia, second-degree atrioventricular block, and ST-segment depression. Laboratory tests showed an elevated white blood cell count (14.29 × 10^9^/L). A preliminary diagnosis of “Type I respiratory failure” was made. Treatment with cefotaxime sodium for anti-infection, as well as antispasmodic, anti-asthmatic, and diuretic symptomatic therapies, yielded no improvement. During hospitalization, the patient experienced recurrent episodes of restlessness and agitation. On the night of May 21, she developed orthopnea, persistent agitation, and hiccups. Arterial blood gas analysis indicated significant deterioration in oxygenation function, prompting urgent transfer to our hospital’s emergency department.

At 06:02 on May 22, the patient experienced an acute disturbance of consciousness, accompanied by tremors in the left limb, trismus, and severe hypoxemia. Immediate endotracheal intubation and mechanical ventilation were performed, followed by transfer to the Intensive Care Unit (ICU). Admission physical examination revealed: body temperature 39.7°C, heart rate 105 beats/minute, respiratory rate 12 breaths/minute (on mechanical ventilation), blood pressure 144/86 mmHg. The patient was conscious. No jaundice or petechiae were observed on the skin or mucous membranes. The extremities were warm with weak pulses. Bilateral pupils were equal, round, and approximately 2.5 mm in diameter, with prompt light reflex. Bilateral breath sounds were coarse without audible moist rales. Cardiac auscultation revealed an irregular heart rhythm with a distinct gallop rhythm. The abdomen was soft and non-tender without rebound tenderness. No edema was present in the lower limbs. Pathological reflexes were not elicited. Multi-organ life support treatment was initiated immediately upon ICU admission.

Cardiovascular system: The patient exhibited significant myocardial injury. The troponin I level increased rapidly from 0.206 μg/L upon admission to 0.804 μg/L on May 23, accompanied by an elevated CK-MB level of 49.8 μg/L (Supplementary Table 1). Bedside echocardiography revealed impaired left ventricular diastolic function, and dynamic electrocardiography recorded frequent sinus pauses (maximum interval of 2.8 s) and second-degree atrioventricular block. After discussion, the patient’s family declined temporary pacemaker implantation. To maintain hemodynamic stability, vasoactive agents including Isoprenaline Hydrochloride Injection and Noradrenaline Bitartrate (maximum dose: 11 μg/kg/min) were administered sequentially, supplemented with Nitroglycerin Injection to improve coronary perfusion. Following active treatment, the myocardial enzyme profile showed improvement compared to previous levels.

**FIGURE 2:**
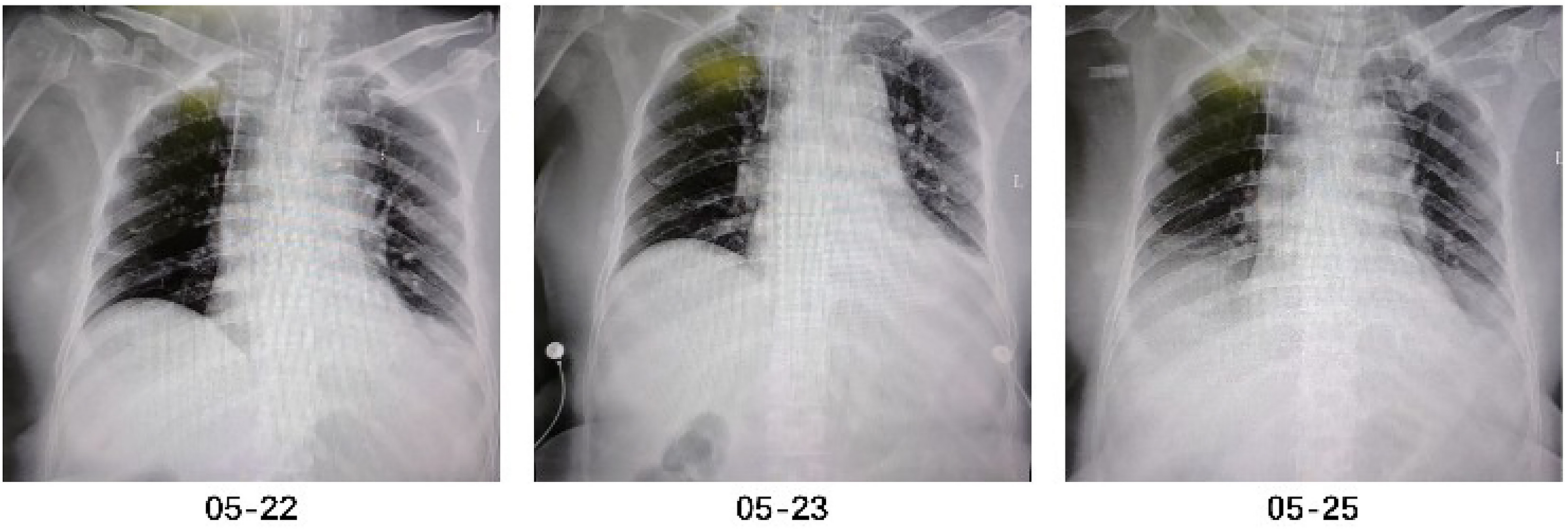
Bedside chest radiographs during hospitalization. The bedside chest radiograph on May 22 showed a small amount of effusion in both lungs and a small amount of pleural effusion on both sides; the radiograph on May 23 showed a small amount of effusion in the left lower lung, a small amount of pleural effusion on the left side, and widening of the right upper mediastinal shadow, which was roughly similar to the radiograph of May 22; the radiograph on May 25 showed a small amount of effusion in both lower lungs and a small amount of pleural effusion on both sides, which was progressive compared to the radiograph of May 23, and widening of the right upper mediastinal shadow, which was similar to the previous radiographs.

Respiratory system: Mechanical ventilation was initiated after admission to the intensive care unit, utilizing the Synchronized Intermittent Mandatory Ventilation (SIMV) mode. Initial settings were FiO2 50%, tidal volume (VT) 420 ml, and positive end-expiratory pressure (PEEP) 6 cmH2O. Parameters were subsequently adjusted dynamically based on daily arterial blood gas analysis results (Supplementary Table 1) and bedside monitoring data. Attempts to switch to the Continuous Positive Airway Pressure (CPAP) mode and perform spontaneous breathing trials (SBTs) on May 24 and 26 failed due to patient intolerance, manifested by increased respiratory rate (>35 breaths/min), inadequate tidal volume (<200 ml), and a sharp decline in oxygen saturation (SpO2 < 88%). Serial bedside chest radiographs demonstrated progressive pulmonary deterioration: an expanding infiltrate in the left lower lobe was noted on May 23, which progressed to diffuse bilateral pulmonary infiltrates accompanied by bilateral pleural effusions by May 26 (Figure 2).

Regarding infection indicators (Supplementary Table 1): The patient’s white blood cell count peaked at 21.28 × 10^9^/L (neutrophil percentage > 90%) on the day of admission. Following empirical antibiotic therapy with meropenem, anti-inflammatory therapy with corticosteroids, and immunomodulation with intravenous immunoglobulin, the count gradually decreased but subsequently rebounded to 13.45 × 10^9^/L on May 25. The procalcitonin level exhibited a continuous decline from 0.51 ng/ml on admission to 0.20 ng/ml. The body temperature demonstrated a remittent fever pattern, fluctuating between 37.5 °C and 39.7 °C during hospitalization (Supplementary Table 1). Multiple blood, urine, and sputum cultures obtained during the ICU stay were negative. Tests for a panel of seven common respiratory viruses and SARS-CoV-2 PCR were also negative (Supplementary Table 2).

**FIGURE 3:**
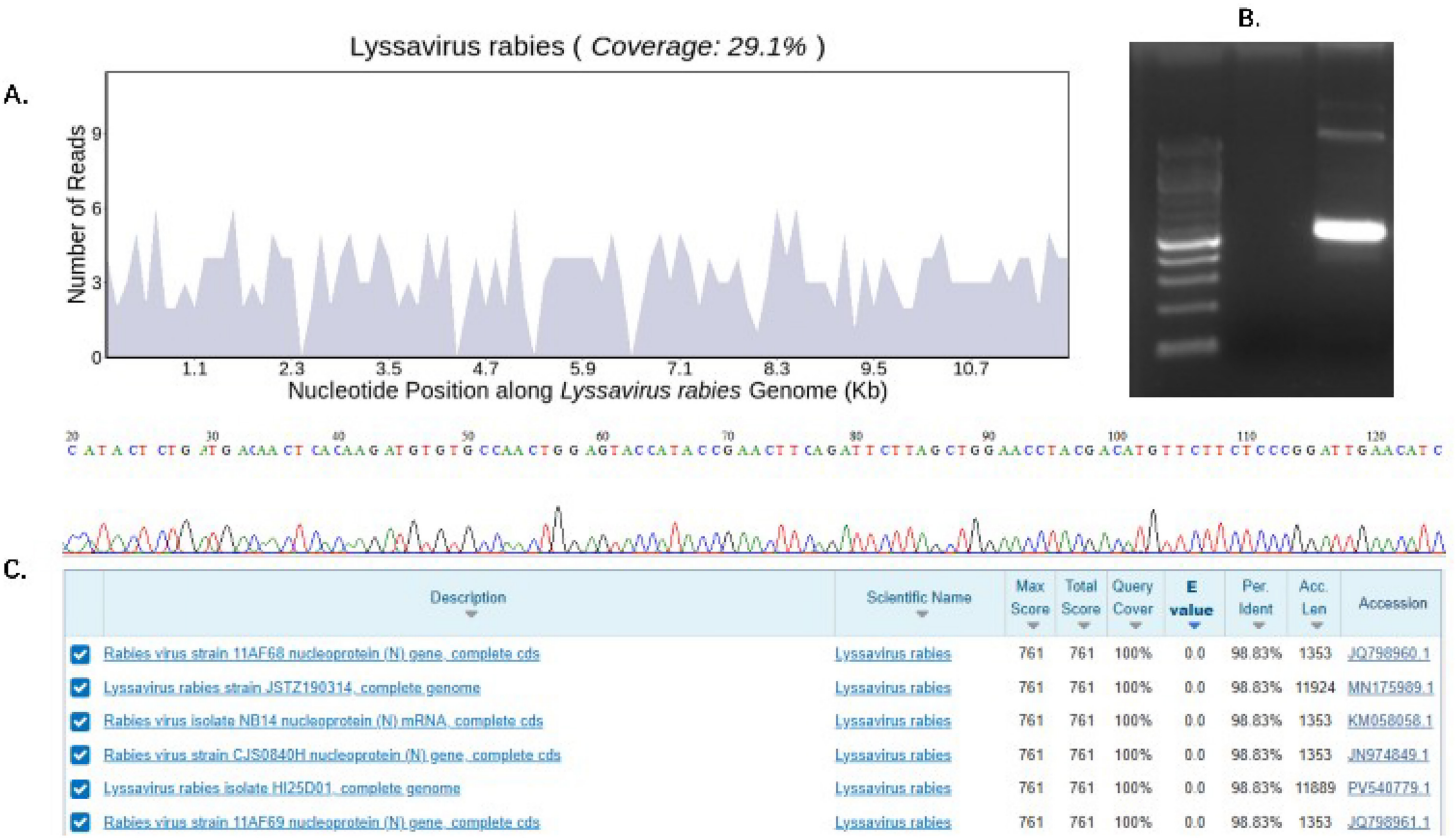
Results of rabies virus targeted next-generation sequencing (tNGS) and PCR validation. **(A)** Rabies virus tNGS result-genome coverage map. **(B)** PCR target band. **(C)** Sanger sequencing: genomic alignment results of the sample PCR product sequence.

The patient’s neurological symptoms demonstrated progressive worsening: On the day following ICU admission (May 23), physical examination revealed mild photophobia (manifested as an enhanced blink reflex to bright light). These symptoms continued to progress. By May 27, photophobia had significantly worsened, presenting as blepharospasm and active avoidance movements in response to bright light, accompanied by a marked increase in oral secretions and significantly elevated muscle tone in all four limbs. After obtaining the written informed consent from the family, a BALF sample was collected on May 29th and sent for tNGS testing. The sample nucleic acids were extracted using automatic nucleic acid extractor Purifier 16 (GENFINE, Beijing, China). The library was constructed using the instructions of VAHTS DNA Clean Beads (Vazyme, Nanjing, China) and Hieff NGS OnePot Flash DNA Library Prep Kit (Yeason BioTech, Shanghai, China). The pathogen was enriched using probes DF_PC2_v1.0.0 (Dinfectome Inc., Nanjing, China). Sequencing was performed on the DNBSEQ-G99 platform (Shenzhen MGI Technology Company) in single-end mode, with each read fragment consisting of 50 base pairs. The detection limit of this process was 50 copies/mL. Quality control and analysis procedures are described in the Supplementary materials. The report was returned within 24 h, and 279 rabies virus sequences were detected (May 30), with a genomic coverage of 29.1%, the relative abundance of 83.78%, and a reference concentration of 32,584 copies/ml (Figure 3A). Subsequent Sanger sequencing in the laboratory further confirmed the presence of rabies virus (RABV) DNA (Table 1 and Figures 3B, C). Initially, the family repeatedly denied any history of animal contact. However, upon further questioning, it was obtained that the patient had contact with domestic puppy 1 week prior to admission, resulting in three non-bleeding scratches on left knee joint that had healed after admission. This information, combined with the progressively worsening neurological symptoms, led to a high clinical suspicion of rabies.

**TABLE 1:** Rabies virus polymerase chain reaction (PCR)-specific primer sequences.

Species	Primer name	Primer sequence (5′–>35′)	Product length
Rabies virus	P510	ATAGAGCAGATTTTCGAGACAGC	455 bp
	P942	CCCATATAACATCCAACAAAGTG	

Despite maximal supportive care in the ICU, including mechanical ventilation, immunomodulatory therapy, and multi-organ support, the patient’s condition continued to deteriorate. On the day the tNGS report was received (May 30), the attending physician repeatedly explained to the family the necessity of continued hospitalization and the life-threatening risks associated with discharge against medical advice. In addition, the patient was transferred to a separate room, and contact isolation measures were performed for related medical staff. The risk of infection (e.g., tetanus) was assessed, and the family members were advised to take isolation precautions. Nevertheless, the family insisted on taking the patient out of the hospital. At the time of discharge, the patient had a Glasgow Coma Scale (GCS) score of 3, indicating deep coma, with sluggish pupillary light reflexes bilaterally. The patient was transferred with the endotracheal tube and all life-support devices in place, and the prognosis was considered extremely poor.

## 3 Discussion

Rabies, as a zoonosis of significant public health importance, presents increasing diagnostic challenges in clinical practice. This case successfully confirmed a rabies infection without a clear exposure history through tNGS technology, providing important insights for discussing diagnostic strategies for atypical rabies.

In the early stages of the routine clinical diagnosis process, this patient did not exhibit typical clinical manifestations, and a head CT scan did not show any obvious signs of rabies. Clinical sample cultures also failed to produce positive results. In particular, the ambiguity regarding exposure fully illustrates the difficulty of diagnosing atypical rabies. In such cases, the combination of atypical clinical presentation and an unclear exposure history directly leads to diagnostic delays (12). This case suggests that for patients with unexplained multi-system damage, especially those with progressive neurological symptoms, the possibility of rabies should be considered even in the absence of a definitive animal contact history.

Compared to traditional diagnostic methods, tNGS demonstrated significant technical advantages in this case. By detecting tNGS in BALF samples, diagnostically significant viral sequences (279 reads, relative abundance 83.78%) were successfully detected in cases where rabies was clinically suspected but could not be confirmed by routine tests. This also highlighted the diagnostic significance and value of probe-capture tNGS for rabies in live patients without typical exposure history. Compared with mNGS, tNGS could reduce sequencing redundancy and costs by targeting and enriching pathogens, which is conducive to its routine application in clinical practice. However, the traditional tNGS technology relies on multiplex pcr technology, which is prone to the limitation of primer quantity and the interference of primer dimers, thereby affecting the amplification of pathogens and sequencing results. tNGS based on probe hybridization capture target pathogens through probes, which do not interfere with each other and can detect up to thousands of pathogens in a single test (13). Therefore, by selecting appropriate target panels based on the clinical manifestations of patients and further using bioinformatics methods, probe-capture tNGS could effectively assist in clinical diagnosis (14).

In this study, we used probe-capture tNGS to effectively detect rabies virus nucleic acid in the patient’s BALF sample. It is noteworthy that cerebrospinal fluid (CSF), saliva, skin biopsy, and serum samples are currently used as diagnostic samples for surviving rabies patients, which is related to the high neurotropism and viral load of the rabies virus (15, 16). In this case, however, the patient’s head CT findings were atypical, and a neurological consultation confirmed that CSF sampling was unnecessary. Furthermore, skin biopsy and saliva samples have more stringent requirements for collection and processing in rabies diagnosis, affecting sensitivity. In contrast, although BALF samples have limitations in terms of viral load, their collection is easier. The probe-capture tNGS we employed can efficiently capture the virus from low-viral-load samples through probe enrichment, enabling the diagnosis of rabies. Our study successfully demonstrated the significant value of respiratory samples and probe-capture tNGS technology in detecting rabies in patients without typical symptoms. However, tNGS is not currently the sole basis for diagnosing or ruling out rabies for rabies virus RNA is a single-stranded negative strand that is easily degraded and has rare lineages. It still needs to be combined with clinical characteristics and standard PCR procedures for comprehensive judgment and interpretation. Future research should focus on evaluating the performance of tNGS in different disease stages and different sample types, establishing standardized detection protocols and interpretation guidelines, and gradually clarifying the applicable criteria for patient testing.

From a public health perspective, this case reveals diagnostic gaps within the current rabies surveillance system. For cases with no clear traceable exposure history, more comprehensive standards for collecting animal contact histories should be established to enhance the identification of atypical cases, particularly through systematic screening of non-classical exposure routes such as minor skin injuries and mucosal exposures. Meanwhile, the application of tNGS provides a novel tool for molecular epidemiological studies of rabies. Whole-genome sequencing of the virus enables precise tracing of transmission chains, which is of significant value evaluating the vaccine’s protective efficacy, formulating regional prevention and control strategies, and monitoring the disease. Efforts should be made to promote the standardized application of such new technologies.

## 4 Summary

To our knowledge, this is among the first reports utilizing probe-capture tNGS of BALF to diagnose human rabies with unclear exposure. However, its widespread application still faces practical challenges such as technical barriers and standardization of application. Future work should focus on further refining testing standards and application guidelines to promote the rational use of this technology in the field of rabies diagnosis.

## Data Availability

The original contributions presented in this study are included in this article/Supplementary material, further inquiries can be directed to the corresponding author.
