# Supplementary material for: Targeted next-generation sequencing confirms rabies in a patient with unknown exposure: a case report
Source: Front Med (Lausanne). 2026 Apr 1;13:1766261. doi: 10.3389/fmed.2026.1766261 (PMC13078974; doi:10.3389/fmed.2026.1766261)
Supplement: Supplementary file 1 [file Supplementary_file_1.docx]

**Supplementary materials for**

**Targeted next-generation sequencing confirms rabies in a patient with unknown exposure: a case report**

**1 Quality control process**

The quantity and quality were assessed using Qubit 4.0 Fluorometers (Thermo Fisher Scientific, Waltham, MA, USA) and Pico488 dsDNA quantification reagent (Lumiprobe, Hunt Valley, MD, USA), ensuring sufficient concentration and purity for library preparation.

Library quality was confirmed by Agilent 2100 Bioanalyzer system (Agilent Technologies, Santa Clara, CA, USA) and the Qubit dsDNA HS Assay Kit (Thermo Fisher Scientific, Waltham, MA, USA).

The entire experimental process included the simultaneous use of positive, negative, blank, and weakly positive control samples to rule out contamination and interference from other samples, thus ensuring the accuracy of the test.

**2 Bioinformatics**

Raw sequencing data were split by bcl2fastq2 (version 2.20, Illumina, San Diego, CA, USA) and were filtered by Trimmomatic (v0.36) to remove low-quality reads and adapter. Host sequences were subtracted by mapping to the human reference genome (hs37d5) using Bowtie2 (version 2.2.6). Remaining reads were aligned to a microorganism genome database (download from GenBank release 238, <https://ftp.ncbi.nlm.nih.gov/genbank/>). Microbial identification was performed using Kraken (version 2.0.7).

**Table S1. Trends in Daily Body Temperature, Inflammatory Markers, and Arterial Blood Gases During Hospitalization.** This figure summarizes daily laboratory values and arterial blood gas indices from May 22 to May 30 in a 68-year-old woman later confirmed to have rabies. Core parameters include body temperature (°C), white blood cell count (WBC, ×10^9^/L), neutrophil percentage (%), lymphocyte percentage (%), monocyte percentage (%), absolute neutrophil count (ANC, ×10^9^/L), absolute lymphocyte count (ALC, ×10^9^/L), absolute monocyte count (AMC, ×10^9^/L), red blood cell count (RBC, ×10^12/L), hematocrit (Hct, %), platelet count (Plt, ×10^9^/L), C-reactive protein (CRP, mg/L), and arterial blood gas metrics including pH, PaCO2 (mmHg), PaO2 (mmHg), HCO3^−^ (mmol/L), standard bicarbonate (SBC, mmol/L), base excess in blood (BE-B, mmol/L) and extracellular fluid (BE-ecf, mmol/L), arterial oxygen saturation (SaO2/SpO2, %), PaO2/FiO2 ratio (P/F, mmHg), anion gap (AG, mmol/L), lactate (Lac, mmol/L), and electrolytes K^+^, Na^+^, Cl^−^, Ca^2+^, Mg^2+^ (all in mmol/L). Trends show persistent leukocytosis with neutrophilia (peak WBC 20.1×10^9^/L on May 22), fluctuating temperature (36.2–38.8°C), progressive hypoxemia with reduced P/F ratio (lowest 143.9 mmHg on May 25), hypercapnia with compensated metabolic alkalosis features (elevated HCO3^−^ and base excess over time), and intermittent hypernatremia (peak Na^+^ 161.5 mmol/L on May 29). BALF for targeted next-generation sequencing (tNGS) was collected on May 29, and rabies virus (RABV) was detected on May 30. Abbreviations: BALF, bronchoalveolar lavage fluid; tNGS, targeted next-generation sequencing; RABV, rabies virus; ICU, intensive care unit.

| **Laboratory Test** | **05-22** | **05-23** | **05-24** | **05-25** | **05-26** | **05-27** | **05-28** | **05-29** | **05-30** |
| --- | --- | --- | --- | --- | --- | --- | --- | --- | --- |
| **Temp (°C)** | 38.8 | 37 | 38.2 | 37 | 36.7 | 36.2 | 37.2 | 37.8 | 37.5 |
| **WBC (×10⁹/L)** | 20.1 | 18.53 | 15.42 | 13.22 | 11.78 | 9.81 | 13.45 | 12.24 | 16.63 |
| **Neutrophils (%)** | 93.1 | 94.8 | 94.7 | 92.3 | 91.8 | 92.6 | 90.2 | 87.3 | 90.3 |
| **Lymphocytes (%)** | 4.6 | 4.1 | 3 | 4.8 | 4.9 | 4.1 | 5.1 | 5.9 | 6.3 |
| **Monocytes (%)** | 2.3 | 1.1 | 2.3 | 2.9 | 3.3 | 3.2 | 4.3 | 6.8 | 3.3 |
| **ANC (×10⁹/L)** | 18.71 | 17.57 | 14.6 | 12.2 | 10.81 | 9.08 | 12.13 | 10.69 | 15.02 |
| **ALC (×10⁹/L)** | 0.92 | 0.76 | 0.46 | 0.63 | 0.58 | 0.4 | 0.73 | 0.72 | 1.05 |
| **AMC (×10⁹/L)** | 0.46 | 0.2 | 0.35 | 0.38 | 0.39 | 0.31 | 0.58 | 0.83 | 0.55 |
| **RBC (×10¹²/L)** | 4.88 | 4.26 | 4.18 | 4.34 | 4.3 | 4.16 | 4.74 | 4.68 | 4.3 |
| **Hct (%)** | 0.44 | 0.39 | 0.39 | 0.4 | 0.4 | 0.38 | 0.44 | 0.45 | 0.41 |
| **PIt (×10⁹/L)** | 332 | 257 | 219 | 212 | 253 | 180 | 238 | 291 | 291 |
| **CRP (mg/L)** | 10.57 | 8.32 | 4.9 | - | 1.49 | - | - | 3.81 | 3.49 |
| **pH** | 7.307 | 7.208 | 7.321 | 7.364 | 7.358 | 7.391 | 7.323 | 7.329 | 7.386 |
| **PaCO₂ (mmHg)** | 45.6 | 61.6 | 52.1 | 55.3 | 63.7 | 53 | 62.1 | 60.5 | 55.6 |
| **PaO₂ (mmHg)** | 89.5 | 93.7 | 74.2 | 64.7 | 77.5 | 61.6 | 75.8 | 74.4 | 75.3 |
| **HCO₃⁻ (mmol/L)** | 23 | 24.7 | 27.2 | 31.8 | 36.2 | 32.4 | 32.5 | 32.2 | 33.6 |
| **SBC (mmol/L)** | 21.9 | 20.7 | 24.8 | 30.1 | 32.8 | 30.1 | 28.1 | 28.1 | 31.1 |
| **BE-B (mmol/L)** | -2.9 | -4.5 | 0.6 | 6.4 | 9.2 | 6.5 | 4.4 | 4.3 | 7.4 |
| **BE-ecf (mmol/L)** | -3.5 | -3.4 | 0.9 | 6.3 | 10.5 | 7.3 | 6.3 | 6 | 8.4 |
| **SaO₂/SpO₂ (%)** | 95.7 | 95.2 | 93 | 90 | 93.8 | 89.9 | 93.2 | 93 | 94.1 |
| **P/F ratio (mmHg)** | 178.9 | 187.5 | 164.9 | 143.9 | 172.3 | 176.1 | 189.5 | 186.1 | 157 |
| **AG (mmol/L)** | 15.2 | 6.6 | 9.7 | 6.7 | 6.7 | 6.7 | 9.8 | 15.1 | 12.8 |
| **Lac (mmol/L)** | 1.4 | 3.5 | 2.4 | 2 | 2.4 | 3.1 | 3.2 | 3.7 | 2.8 |
| **K⁺ (mmol/L)** | 3.3 | 4.3 | 4.2 | 3.9 | 4.1 | 3.7 | 3.5 | 3.8 | 4 |
| **Na⁺ (mmol/L)** | 148.3 | 144.5 | 145 | 146.9 | 145.9 | 143.7 | 149.9 | 161.5 | 156 |
| **Cl⁻ (mmol/L)** | 110 | 113 | 108 | 108 | 103 | 105 | 108 | 114 | 110 |
| **Ca²⁺ (mmol/L)** | 1.32 | 1.13 | 1.26 | 1.37 | 1.35 | 1.27 | 1.18 | 1.25 | 1.33 |
| **Mg²⁺ (mmol/L)** | 0.95 | 0.49 | 0.69 | 0.83 | 0.9 | 0.77 | 0.76 | 0.79 | 1.14 |

**Table S2. Clinical pathogen detection and results.** Blood, urine, and sputum samples were collected from the patient between May 22 and May 27 for culture. Urine and sputum cultures showed no bacterial growth after two days, and blood cultures showed no bacterial growth after five days. On May 23, the patient underwent respiratory virus panel testing (including influenza A and B viruses, parainfluenza virus types I, II, and III, respiratory syncytial virus, and adenovirus), all of which were negative for seven respiratory viruses. On the same day, a SARS-CoV-2 PCR test was conducted, and the result was negative.

| **Methods** | **Results** |
| --- | --- |
| Culture ^a^ | Neg |
| Respiratory virus testing ^b^ | Neg |
| SARS-CoV-2 testing | Neg |

^a^ Blood culture, urine culture, and sputum culture.

^b^ Respiratory virus testing included influenza A and B viruses, parainfluenza virus types I, II, and III, respiratory syncytial virus, and adenovirus.
